# Decreased Corticospinal Excitability after the Illusion of Missing Part of the Arm

**DOI:** 10.3389/fnhum.2016.00145

**Published:** 2016-04-14

**Authors:** Konstantina Kilteni, Jennifer Grau-Sánchez, Misericordia Veciana De Las Heras, Antoni Rodríguez-Fornells, Mel Slater

**Affiliations:** ^1^Event Lab, Department of Personality, Evaluation and Psychological Treatment, University of BarcelonaBarcelona, Spain; ^2^IR3C Institute for Brain, Cognition, and Behaviour, University of BarcelonaBarcelona, Spain; ^3^Cognition and Brain Plasticity Group, Bellvitge Biomedical Research Institute, L’Hospitalet de LlobregatBarcelona, Spain; ^4^Department of Basic Psychology, L’Hospitalet de Llobregat, University of BarcelonaBarcelona, Spain; ^5^Neurophysiology Section, Neurology Service, Hospital Universitari de Bellvitge, Bellvitge Biomedical Research InstituteBarcelona, Spain; ^6^Institució Catalana de Recerca i Estudis AvançatsBarcelona, Spain

**Keywords:** illusory amputation, body ownership, corticospinal excitability, transcranial magnetic stimulation, virtual reality

## Abstract

Previous studies on body ownership illusions have shown that under certain multimodal conditions, healthy people can experience artificial body-parts as if they were part of their own body, with direct physiological consequences for the real limb that gets ‘substituted.’ In this study we wanted to assess (a) whether healthy people can experience ‘missing’ a body-part through illusory ownership of an amputated virtual body, and (b) whether this would cause corticospinal excitability changes in muscles associated with the ‘missing’ body-part. Forty right-handed participants saw a virtual body from a first person perspective but for half of them the virtual body was missing a part of its right arm. Single pulse transcranial magnetic stimulation was applied before and after the experiment to left and right motor cortices. Motor evoked potentials (MEPs) were recorded from the first dorsal interosseous (FDI) and the extensor digitorum communis (EDC) of each hand. We found that the stronger the illusion of amputation and arm ownership, the more the reduction of MEP amplitudes of the EDC muscle for the contralateral sensorimotor cortex. In contrast, no association was found for the EDC amplitudes in the ipsilateral cortex and for the FDI amplitudes in both contralateral and ipsilateral cortices. Our study provides evidence that a short-term illusory perception of missing a body-part can trigger inhibitory effects on corticospinal pathways and importantly in the absence of any limb deafferentation or disuse.

## Introduction

Body ownership illusions refer to a class of perceptual illusions where an external object is experienced as part of, or even as one’s entire own body under certain multisensory/sensorimotor conditions (Tsakiris, 2010; Ehrsson, 2012; Kilteni et al., 2015). The most classic example is the rubber hand illusion (RHI) where participants see a rubber hand in front of them that is being touched at the same time and at homologous body areas as their real unseen hand. After some seconds of such spatiotemporally congruent visuotactile stimulation, the participants experience the illusion that the rubber hand is their real hand (Botvinick and Cohen, 1998; Ehrsson et al., 2004). Using similar methodology, participants can experience the illusion of owning an artificial body when they see it from a first person visual perspective (1PP) and while this is being touched in correspondence with their real body (Petkova and Ehrsson, 2008; Maselli and Slater, 2013). Besides the subjective experience, it has been shown that these illusions produce physiological changes on the real body; owning a fake hand decreases the temperature (Moseley et al., 2008; Hohwy and Paton, 2010) and changes the temperature sensitivity of the real hand (Llobera et al., 2013), increases the histamine reactivity in the real arm (Barnsley et al., 2011), slows the tactile processing (Moseley et al., 2008) and attenuates tactile detection performance on the real hand (Zopf et al., 2011). Additionally, a threat to the fake body while experiencing the illusion elicits brain activity in insula and anterior cingulate cortex that is comparable to that triggered when the real counterpart is threatened (Ehrsson et al., 2007), elicits motor cortex activation (González-Franco et al., 2013), triggers the defensive mechanisms for immediate action (Kilteni et al., 2012), and produces higher skin conductance responses (Petkova and Ehrsson, 2008) and heart rate deceleration (Slater et al., 2010) compared to when the illusion is not experienced.

Experimental studies on body ownership illusions suggest that while a basic resemblance between the non-bodily object and a generic human body is needed (Kilteni et al., 2015), morphological differences with the specific participant’s real counterpart, as for example in terms of skin color (Farmer et al., 2012; Kilteni et al., 2013; Maister et al., 2013; Peck et al., 2013) or age (Banakou et al., 2013), do not inhibit the illusion (Maister et al., 2014). Of particular interest are the cases of body ownership illusions under differences in body integrity. Ehrsson et al. (2008) considered whether upper-limb amputees can experience ownership toward an artificial hand and applied synchronous visuotactile stimulation between the index finger of the rubber hand and points on the amputees’ stump that could elicit phantom sensations on the phantom index finger. Although, the reported illusion was weaker compared to when the amputees’ intact hand was tested, neuroimaging data revealed activation in multisensory brain areas (Schmalzl et al., 2014) that had been previously shown to operate the RHI for non-amputated people (Ehrsson et al., 2004; Brozzoli et al., 2012). Similar illusory sensations were reported under visuotactile stimulation combined with myoelectric motor control of an advanced humanoid robotic prosthesis (Rosén et al., 2009), whereas a full body ownership illusion triggered by visuotactile stimulation was reported toward an intact mannequin (Schmalzl et al., 2011).

Addressing the same question but in the reverse direction, is it possible to induce the illusion of missing an actually present body-part in healthy individuals? In a series of experiments, Guterstam et al. (2010) showed that healthy people could experience strong sensations of having an invisible hand, when correlated visuotactile stimuli were applied between the participants’ occluded hand and the empty space in front of them. Similar sensations of having an invisible, vanished or completely transparent finger were reported when stroking the participants’ ring finger in synchrony with the empty space that corresponded to an invisible ring finger of a rubber hand (Lewis et al., 2012). Analogously, the illusory experience of having phantom or telescoped limbs was induced through a body ownership illusion toward an amputated mannequin (Schmalzl and Ehrsson, 2011). Nevertheless, all the above-mentioned studies aimed at inducing analogs of phantom sensations (i.e., the body-part is there but it is somehow invisible), and this is clearly reflected in the experimental methods, given that tactile stimulation on the real body-part should emphasize its presence rather its absence.

The present study aimed at investigating whether intact individuals can experience an illusory loss of their limb. Such an illusory perception – if at all possible – would theoretically entail a perceptually driven temporary reduction of the sensorimotor capacities and representations in the brain, given that the absence of the limb would mandate its disuse. Neurophysiological studies have shown that perturbations in the somatic afferent and/or motor information evoked by a decreased or no use of a body-part, produce neuroplastic changes in the motor cortex. For example, chronic deafferentation due to limb amputation leads to a reorganization of the motor pathways (Cohen et al., 1991) and cortical representations (Pascual-Leone et al., 1996) in the deafferented hemisphere. Transient deafferentation through anesthetic block of a body region induces an increase in the cortical excitability of the motor pathways for the muscles immediately proximal to the deafferented body area (Brasil-Neto et al., 1992), an increase in the excitability of the homotopic sites in the ipsilateral hemisphere (Werhahn et al., 2002) and a reduction in the cortical representation of the muscles that are encompassed by the anesthetized area and deprived from their normal sensory input (Rossini et al., 1996). Furthermore, limb disuse due to restriction of movement (i.e., immobilization) for several hours, days, or weeks has been shown to produce a decrease in the cortical thickness of the contralateral sensorimotor cortex (Langer et al., 2012), a decrease in the motor cortex area size (Liepert et al., 1995) and in the cortical excitability of the immobilized muscle (Facchini et al., 2002; Huber et al., 2006; Avanzino et al., 2011; Ngomo et al., 2012). On the basis of these experimental results, it could be speculated that an illusory amputation would drive a decrease in the excitability of the sensorimotor cortex that is contralateral to the amputation side.

Using immersive virtual reality (VR; Spanlang et al., 2014), all participants were shown a virtual body from a 1PP which for half of them, appeared as if amputated in its right upper limb. Single pulse transcranial magnetic stimulation (TMS) was applied before and after the experiment on both right and left primary motor cortices, and motor evoked potentials (MEPs) from two muscles associated with the amputation area were recorded from each hand. We expected that participants would experience the virtual body as their own body, and that those in the amputation condition would experience the illusion of missing part of their right arm. Moreover, we hypothesized that the illusory experience of missing part of the right arm would yield changes in the corticospinal excitability of the associated left hemisphere. Specifically, we speculated that the post-VR corticospinal excitability of the left hemisphere (right hand) would decrease compared to the pre-VR one. No changes were expected in the right hemisphere and in neither of the hemispheres of the control participants.

## Materials and Methods

### Participants

Forty-nine right-handed participants with no previous history of epileptic episodes or neurological disorders participated in the study. The study was approved by the Comitè Ètic d’ Investigació Clínica and it was conducted in the Bellvitge University Hospital of Barcelona in Spain. The procedures for the TMS protocol accomplished the corresponding safety guidelines (Rossi et al., 2009). All participants gave written informed consent and were compensated with 30€ for their participation after the end of the experiment.

Nine participants were excluded because of technical problems during either the TMS or the VR session. Data analysis was therefore based on the data of 40 participants. Before the experiment basic demographic details were recorded for all participants and they completed the Edinburgh handedness questionnaire (Oldfield, 1971). Given previous evidence that personality characteristics may influence the experience of body illusions (Kilteni et al., 2013), all participants were further asked to fill in the Spanish version of the NEO-FFI personality inventory (Costa and McCrae, 1992; **Table 1**).

**Table 1:** Demographic details of the participants.

Condition	Amputation	Control
# Participants	20	20
# Females	10	10
# Right-handed	20	20
Age (mean ± SD)	23.50 ± 4.23	24.25 ± 4.47
Neuroticism (mean ± SD)	18.20 ± 8.20	18.95 ± 8.17
Extraversion (mean ± SD)	34.20 ± 6.73	30.55 ± 8.17
Openness to Experience (mean ± SD)	32.75 ± 6.57	33.25 ± 6.04
Agreeableness (mean ± SD)	27.45 ± 6.43	27.75 ± 7.45
Conscientiousness (mean ± SD)	32.70 ± 8.84	31.15 ± 6.99


*There were no significant differences in terms of age or any personality characteristics between the participants of the two conditions.*

### Transcranial Magnetic Stimulation

Transcranial magnetic stimulation was applied over the primary motor cortex under a single pulse protocol. A 70 mm figure-of-8 coil attached to a Magstim Rapid 2 Stimulator (Magstim Company, Carmathenshire, Wales) was used to elicit MEPs from the first dorsal interosseous (FDI) and the extensor digitorum communis (EDC) for each hand. For each pulse, the electromyographic (EMG) activity was recorded through surface Ag/AgCl disk electrodes in a belly tendon montage from both contralateral FDI and EDC muscles, for a total of 700 ms including a 100 ms pre-stimulus window (Medelec Synergy, Oxford Instruments, Pleasantville, NY, USA). The EMG signal was sampled at 5 KHz and band-pass filtered at 1–1000 Hz. All data were exported for off-line analysis using specialized software (Matlab, MathWorks, Natick, MA, USA).

During both TMS sessions, participants wore an elastic lycra fabric cap, on which a 10 cm × 10 cm grid centered on the vertex (Cz position of the international 10/20 EEG positioning system) was drawn in order to allow simple identification of stimulation coordinates for sites separated by 1 cm each. Midline points of the grid were placed 7 cm anterior and 3 cm posterior to the vertex, and from each midline point 10 points separated by one cm were distributed laterally for each hemisphere. During the registrations, the TMS coil was positioned tangentially to each site, with the handle pointing backward (in a lateral to medial and caudal to rostral position) ∼45° lateral from the midline.

### Virtual Reality

During the session of VR, participants were fitted with a Sensics zSight 60 Head Mounted Display (HMD) with embedded headphones. Two mechanical coin type vibrators (model C1034-50L, 1200 ± 300 r.p.m., Shenzhen Linglong Electronics, Co.) connected to an Arduino microcontroller were attached on the participants’ right and left carpal respectively. Two unmediated auditory recordings from a triggered vibrator on human skin (*vibratorSkin*) or on a wooden table (*vibratorTable*) were used. All virtual models were modeled in 3D Studio Max 2010 and 3D Studio Max 2012. The virtual environment was implemented on the XVR platform (Tecchia, 2010) and the virtual body was displayed using a hardware accelerated avatar library (HALCA; Gillies and Spanlang, 2010).

### Procedures

The experiment was a between-groups study with one single factor, the appearance of the body, with two levels: amputated or intact arm. Upon arrival, participants were randomly assigned to the amputation or control condition.

The experimental procedures included one VR session and two TMS sessions (one before and one after the VR), lasting 90 min. During all sessions, participants were seated in a chair, with their legs extended in a horizontal position on top of another support in order to feel comfortable. Both their arms rested on a table in front of them, while their hands were hanging out of the front edge of the table. A box was placed on top of the table in order to conceal the participants’ forearms and hands from view. Participants were told that for the rest of the experiment, they could freely move their head but they were requested to keep their body and especially their arms motionless.

#### TMS Sessions

The excitability of the corticospinal pathway was addressed for each hemisphere. In the pre-VR TMS session and before any registration, the grid location where the highest MEP of the FDI was elicited (Hot Spot) was detected and recorded for each hemisphere (Rossi et al., 2009). Following, the resting motor threshold (RMT), defined as the lowest stimulus intensity needed to evoke a visible MEP > 50 μv in 50% of 10 trials from the relaxed muscle, was explored in the Hot Spot (Rossini et al., 1994).

For each hemisphere, the MEP was assessed in the Hot Spot of the FDI. Ten MEPs were elicited and recorded for each muscle (FDI and EDC) in the FDI Hot Spot at 120% of the RMT. Since the registration of the MEPs for both muscles was done simultaneously after each pulse on each hemisphere, the EDC MEPs were also triggered through stimulating the FDI Hot Spot at 120% of the FDI RMT. The order of the assessed hemispheres was counterbalanced. Throughout both TMS sessions, subjects had to fixate on a marker placed on the wall, 2 m approximately in front of them.

#### VR Session

**FIGURE 1:**
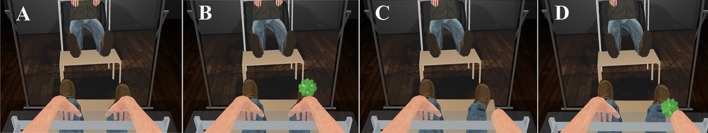
**The participant’s view from the HMD.** **(A)** For both groups, a gender-matched virtual body was seen from 1PP with the same posture with the real body, as if they were spatially coincident. Participants were asked to look around and describe what they see (1 min). **(B)** For both groups, a virtual ball touched the right virtual hand various times while the real hand was physically touched at the same timings (2 min). **(C)** For the amputation group, part of the right virtual arm disappeared, as if amputated (3 min). **(D)** Following, the virtual ball touched the area previously occupied by the right virtual hand and part of the forearm (i.e., the table surface) without triggering any physical touch to the participant’s right hand (10 min).

The session started for both groups identically. Through the HMD, participants saw a complete gender-matched virtual body from a 1PP, spatially coincident with their real body. In order to make the participants’ posture as comfortable as possible while being motionless, a virtual mirror was placed just opposite so that they could see the virtual body both when looking down but also when looking straight ahead (**Figure 1A**). Participants were asked to move their head and describe what they saw around themselves, including the virtual body (1 min). Once the scene was described, the experimenter asked them to focus on the virtual hands for the remainder of the session by looking either directly or through the mirror. Then, a virtual ball appeared and tapped the right virtual carpal, bouncing between the virtual hand and the virtual mirror in random velocities for 2 min (**Figure 1B**). Every time the ball made contact with the virtual carpal and for the whole duration of this contact, the vibrator on the participants’ right hand was triggered and the *vibrationSkin* sound file was reproduced. Therefore, all seen and heard tactile events on the virtual hand were temporally registered with physical touch on the real hand.

The remaining procedures differed between the two conditions in the appearance of the virtual body. For the amputation condition, part of the virtual arm disappeared and the virtual body appeared as if missing part of its right arm (3 min; **Figure 1C**). For the following 10 min, the virtual ball appeared again but it now bounced between the virtual mirror and the table, landing always at the former position of the virtual hand (**Figure 1D**). There was no physical vibration on the real right hand and the sound that accompanied the seen collision of the ball was the *vibrationTable* one. This last phase was inserted to emphasize the absence of the virtual hand: besides the visual input that the body-part was no longer there, additional visual and auditory evidence from the bouncing ball emphasized that expected sensations were not experienced. In contrast, the control participants kept seeing the intact virtual body for 3 min (**Figures 1A,B**) and for the following 10 min, when the virtual ball touched the virtual hand, there was no physical vibration and the sound was changed to *vibrationTable*. All together, both groups received the exact same sensory events with the only difference being the presence or the absence of part of the right virtual arm.

The VR session lasted 16 min. When the session finished, the HMD was removed, participants were reminded to keep their hands motionless and the post-VR TMS session began immediately.

#### Post-experiment Session

When the second TMS session finished, participants were asked to complete a questionnaire with respect to their experience. Then, the purpose of the experiment was explained and they were paid. For ethical purposes, all participants of the amputation condition were asked again to don the HMD and were shown the virtual body with an intact right arm. Finally, all subjects were contacted few weeks after the experiment and they were asked about having any thoughts or sensations concerning the experiment. All of them found the study interesting and none reported any negative feelings or thoughts.

### Response Variables

#### Questionnaire

The post-experiment questionnaire consisted of 19 statements (**Table 2**). We expected that participants of the amputation condition would give higher ratings to the statements on amputation compared to the control subjects, while high scores without significant differences were expected for arm and full body ownership. Low ratings were expected from the control items in both groups and exploratory items with respect to temperature, pain, numbness, and movement related sensations were also included.

**Table 2:** Statements related to the VR experience.

Item statement	Item tag	Component
I felt as if I had a part of my right arm missing	*missright*	*Amputation*
I felt as if I had no right hand	*noright*	
I felt as if I had only one hand	*onehand*	
I felt as if I couldn’t tell where my right hand was	*tellright*	
I felt my right hand less vivid than normal	*lessvivid*	

I felt as if the virtual body were my body	*ownbody*	*Ownership*
I felt as if the right virtual arm were my own arm	*ownarm*	

I felt as if I had two right hands	*tworight*	*Control*
I felt as if I had parts of both of my arms missing	*missboth*	
I felt as if I had a part of my left arm missing	*missleft*	

I felt my right hand cooler than normal	*coolright*	*Temperature*
I felt my left hand cooler than normal	*coolleft*	
I felt my right hand warmer than normal	*warmright*	
I felt my left hand warmer than normal	*warmleft*	

I felt as if my right hand was in pain/was hurting me	*painright*	*Pain*
I felt as if my left hand was in pain/was hurting me	*painleft*	

I felt numb sensations from my right hand or arm	*numbright*	*Numbness*
I felt numb sensations from my left hand or arm	*numbleft*	

I felt as if I couldn’t move my right hand if I wanted to (like being paralyzed)	*nomoveright*	*Movement*


#### MEP Amplitudes

Peak-to peak amplitudes of the MEPs in *μV* were extracted from the EMG registrations (Rossini et al., 1994) using Matlab routines, and after careful visual inspection. As stated above, 10 MEPs were recorded for each hemisphere and muscle, before and after the VR session, resulting to 80 registrations per participant and 320 sets of 10 registrations in total. There were 133 (4.1%) bad registrations (i.e., due to poor signal quality or no observable MEPs). The analysis was performed on 314 registrations (six missing values).

Outlier detection was computed by calculating the mean MEP amplitude for each muscle, hemisphere, time and condition per participant. MEP amplitude values exceeding two standard deviations from the mean were considered as outliers and discarded (Nogueira-campos et al., 2014). Based on this criterion, less than 6% of the trials were discarded from the analyses (181 discarded trials out of 3067 valid trials). After removing the outliers, MEP amplitudes were normalized by applying a logarithmic transformation (Tremblay et al., 2001).

### Statistical Analysis

Statistical analysis was performed with Stata 2013. Between groups differences in questionnaires were compared with Mann–Whitney test. MEP amplitudes were analyzed through two mixed full factorial 2 × 2 × 2 ANOVAs, one per each muscle, with condition as between groups factor, and time and hemisphere as within groups factors. For each *post hoc t*-test, confidence intervals together with the corresponding *p*-value are reported. Residual errors were assessed for normality using the Shapiro–Wilk test. Correlations were evaluated using the Spearman correlation coefficient (ρ).

## Results

### Questionnaire

**FIGURE 2:**
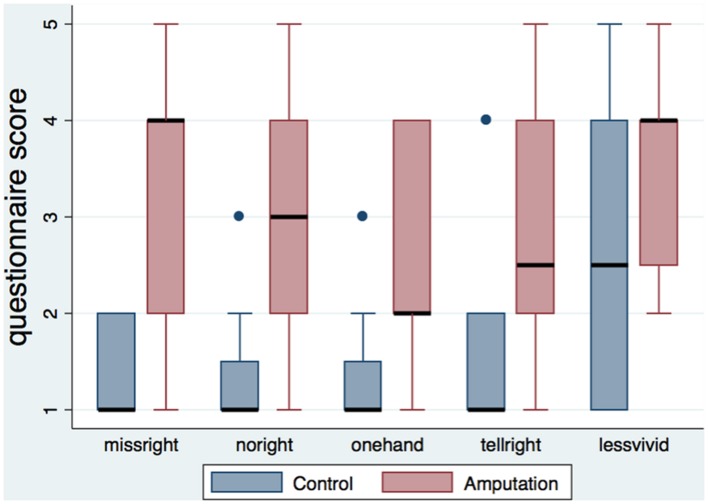
**Boxplot of the amputation related statements per condition.** The height of the boxes represents the interquartile ranges, the black lines represent the medians and the blue dots represent the outliers. Participants in the amputation condition rated the statements related to the illusion of amputation significantly higher than the control group.

**Table 3** shows the medians and interquartile ranges of the questionnaire responses for the two conditions. Participants of the amputation condition rated the amputation statements (*missright*, *noright*, *onehand*, *tellright*, *lessvivid*) significantly higher than the controls (**Figure 2**). No differences were found in arm and full body ownership (*ownarm*, *ownbody*) between the conditions, with both median scores being high (four out of five). No other significant differences were detected, besides the statement about numb feelings on the left hand, where participants in the amputation condition reported significantly less numb sensations for their left hand compared to controls.

**Table 3:** Medians and interquartile ranges of the questionnaire responses for the Control and Amputation conditions.

Statement	Amputee (*n* = 20)		Control (*n* = 20)		Mann–Whitney	Component
				
	Median	IQR	Median	IQR	*p*-value	
*missright*	4	2	1	1	<0.0001	*Amputation*
*noright*	3	2	1	0.5	<0.0001	
*onehand*	2	2	1	0.5	<0.001	
*tellright*	2.5	2	1	1	0.002	
*lessvivid*	4	1.5	2.5	3	0.011	

*ownbody*	4	1	4	2	0.125	*Ownership*
*ownarm*	4	1	4	2	0.386	

*tworight*	1	0.5	1	1	0.677	*Control*
*missboth*	1	0.5	1.5	1	0.106	
*missleft*	1	0	1	1	0.073	

*coolright*	2	2	1.5	1.5	0.624	*Temperature*
*coolleft*	1.5	1	1.5	2	0.725	
*warmright*	2	2.5	2	3	0.843	
*warmleft*	2	1	2	1	0.860	

*painright*	1	0.5	1	1	0.234	*Pain*
*painleft*	1	0.5	1	1	0.388	

*numbright*	2.5	3	3	2.5	1.000	*Numbness*
*numbleft*	1.5	1	3	2.5	0.023	

*nomoveright*	3	2	2	2.5	0.162	*Movement*


*The statements were rated in a 1 to 5 Likert scale where 1 corresponded to ‘strong disagreement’ and 5 to ‘strong agreement.’*

**FIGURE 3:**
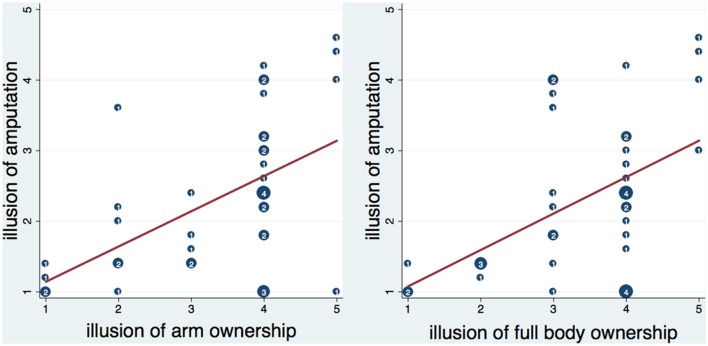
**Scatterplot of the amputation illusion with arm and full body ownership illusion.** Numbers inside circles indicate the number of overlapping points and the red lines represent the fitted regression lines. The amputation illusion was significantly correlated with arm and full body ownership illusion (see text). Moreover, it was significantly predicted by arm ownership (*b* = 0.49, *t* = 3.86, *p* < 0.001) and full body ownership (*b* = 0.52, *t* = 3.52, *p* = 0.001) respectively. Both arm and full body ownership explained a significant proportion of variance in the amputation illusion [arm ownership – *R*^2^ = 0.26, *F*_(1,38)_ = 14.86, *p* < 0.001; full body ownership- *R*^2^ = 0.23, *F*_(1,38)_ = 12.42, *p* < 0.01].

The *amputation* score, calculated as the average of the five amputation statements, was significantly and positively correlated with arm ownership (*ownarm* – ρ = 0.528, *n* = 40, *p* < 0.001) and full body ownership (*ownbody* – ρ = 0.464, *n* = 40, *p* = 0.002; **Figure 3**), and with feelings of not being able to move the right hand (*nomoveright* – ρ = 0.689, *n* = 40, *p* < 0.0001). No other significant correlations were detected between the amputation score and the rest of the items.

### MEP Amplitudes

**FIGURE 4:**
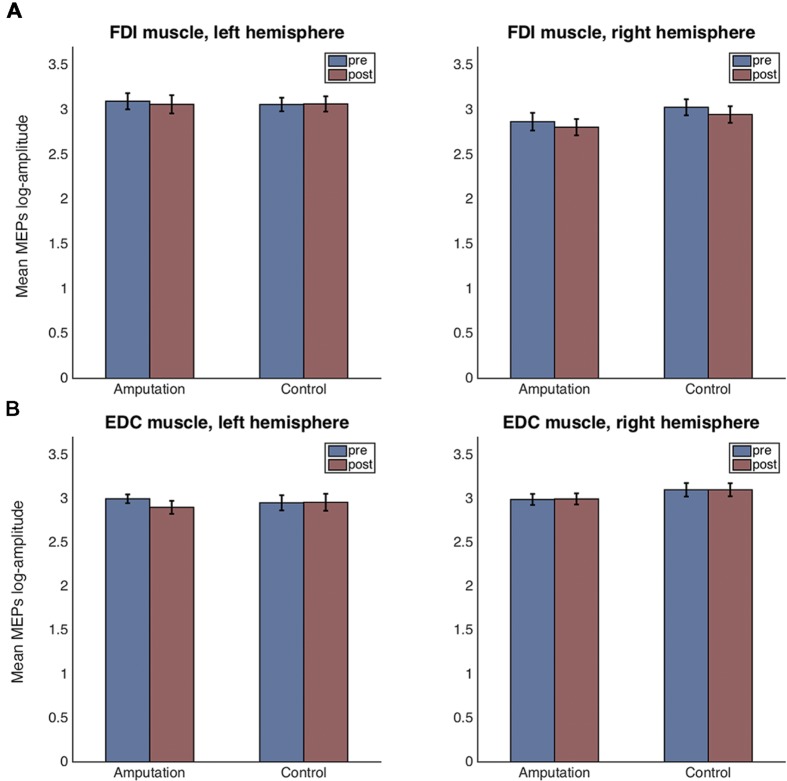
**Mean and standard error plots for the log-transformed MEP amplitudes per hemisphere, time and condition for the FDI muscle **(A)** and for the EDC muscle **(B)****.

**Figures 4A,B** show the means and standard errors for the log-transformed MEP amplitudes per time, hemisphere and condition, for the FDI and EDC muscles respectively.

For the FDI muscle, there was no main effect of condition [*F*_(1,38)_ = 0.42, *p* = 0.523], nor of time [*F*_(1,38)_ = 1.19, *p* = 0.281] but a main effect of hemisphere [*F*_(1,38)_ = 7.81, *p* = 0.008, $\eta_{p}^{2}$ = 0.384] was detected. None of the two or three way interactions were significant. Residuals errors were normally distributed (Shapiro–Wilk test, *p* = 0.502). *Post hoc t*-tests revealed that the FDI amplitudes for the left hemisphere (right hand) were significantly greater than those for the right hemisphere [*t*_(78)_ = 4.84, *p* < 0.001, CI[-0.52, -0.21]].

For the EDC muscle, there was no main effect of condition [*F*_(1,37)_ = 0.45, *p* = 0.507], nor of time [*F*_(1,37)_ = 0.56, *p* = 0.460] but a main effect of hemisphere [*F*_(1,37)_ = 4.76, *p* = 0.035] was detected. None of the two or three way interactions were significant. Residuals errors were not normally distributed (Shapiro–Wilk test, *p* < 0.0001). Visual inspection of the residuals’ plot clearly identified four outlier values corresponding to one participant (**Supplementary Figure S1**). After removal of the outliers, the ANOVA revealed again no main effect of condition [*F*_(1,36)_ = 0.58, *p* = 0.452], nor of time [*F*_(1,36)_ = 0.11, *p* = 0.747] but a main effect of hemisphere [*F*_(1,36)_ = 4.68, *p* = 0.037, $\eta_{p}^{2}$ = 0.370] was detected. None of the two-way interactions were significant. However, the three-way interaction condition × time × hemisphere had significance level *p* = 0.053 [*F*_(1,35)_ = 3.98]. Residual errors were normally distributed (Shapiro–Wilk test, *p* = 0.575). *Post hoc t*-tests for the hemisphere effect revealed that the EDC amplitudes for the left hemisphere (right hand) were significantly lower than those for the right hemisphere [*t*_(74)_ = -4.61, *p* < 0.001, CI[0.13, 0.33]]. For explorative reasons and despite the three-way interaction being non-significant (*p* = 0.053), we performed the *post hoc* comparisons of interest. For the control condition, there were no significant differences in MEP amplitudes before and after the VR session, neither for the left hemisphere [*t*_(18)_ = 0.14, *p* = 0.886, CI[-0.184, 0.212]], nor for the right one [*t*_(17)_ = -0.09, *p* = 0.927, CI[-0.218, 0.199]]. In contrast, for the amputation condition and for the left hemisphere, MEPs after the VR session were significantly smaller compared to those before [*t*_(18)_ = -2.34, *p* = 0.025, CI[-0.426, -0.030]]. No significant differences were observed for the right hemisphere in the amputation condition [*t*_(18)_ = 1.46, *p* = 0.153, CI[-0.055, 0.341]].

### Subjective Experience and MEP Amplitudes

**FIGURE 5:**
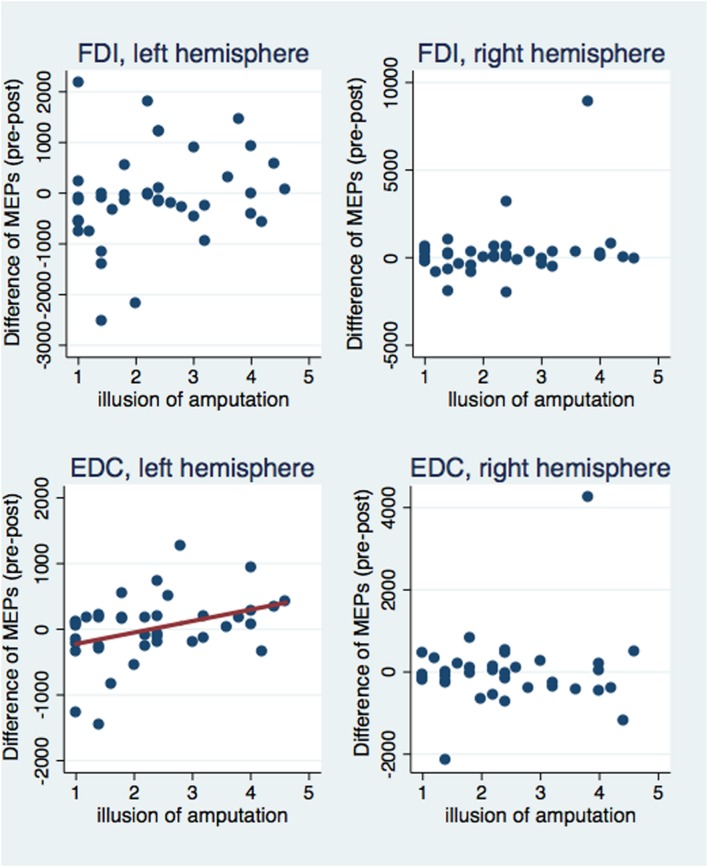
**Scatterplot of the difference between pre and post-VR MEP amplitudes with the illusion of amputation per each muscle and hemisphere.** The difference in amplitudes was significantly correlated with the amputation illusion only for the EDC muscle of the left hemisphere (see text). Similarly, only in this case, the amputation illusion predicted significantly the MEPs’ difference (*b* = 174.33, *t* = 2.53, *p* = 0.016) and explained a significant proportion of its variance [*R*^2^ = 0.12, *F*_(1,37)_ = 6.39, *p* = 0.016].

**FIGURE 6:**
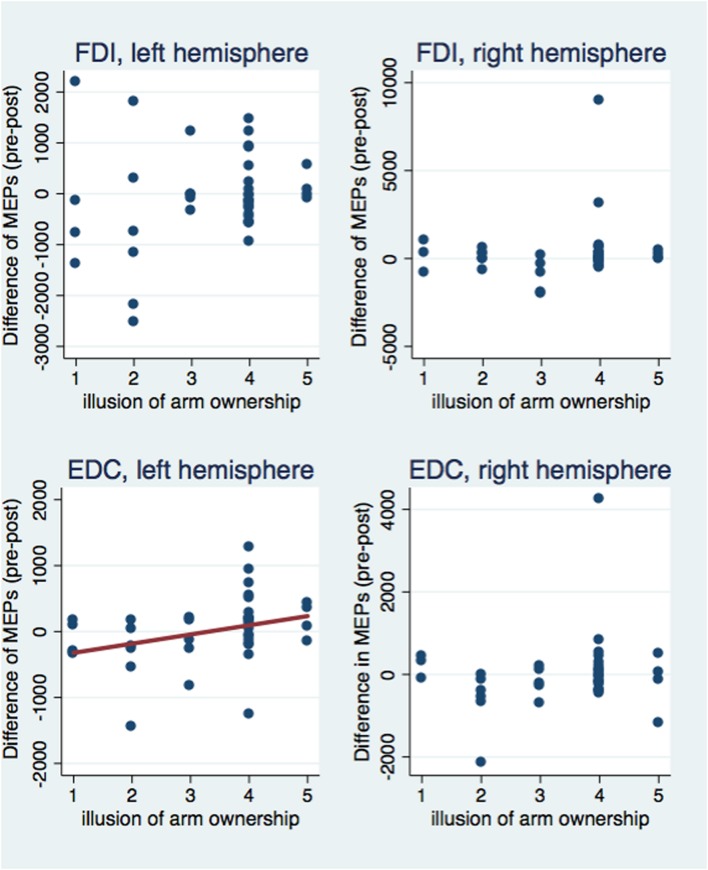
**Scatterplot of the difference between pre and post-VR MEP amplitudes with the illusion of arm ownership per each muscle and hemisphere.** The difference in amplitudes was significantly correlated with the arm ownership illusion only for the EDC muscle of the left hemisphere (see text). Similarly, only in this case, the ownership illusion predicted significantly the MEPs’ difference (*b* = 139.27, *t* = 2.10, *p* = 0.043) and explained a significant proportion of its variance [*R*^2^ = 0.08, *F*_(1,37)_ = 4.40, *p* = 0.043].

Next, we investigated correlations between the subjective illusion of ownership and amputation with the difference between the pre and post-VR MEP amplitudes (**Figures 5** and **6**). The amplitude difference for the FDI muscle of the left hemisphere was not correlated with the illusion of amputation (ρ = 0.250, *n* = 40, *p* = 0.120) or arm ownership (ρ = 0.222, *n* = 40, *p* = 0.168). Similarly, no correlation was found between the FDI amplitudes’ difference of the right hemisphere with amputation (ρ = 0.137, *n* = 39, *p* = 0.407) or arm ownership (ρ = 0.131, *n* = 39, *p* = 0.427). Additionally, the EDC amplitudes’ difference of the right hemisphere was not significantly correlated with the illusion of amputation (ρ = -0.044, *n* = 38, *p* = 0.795) and arm ownership (ρ = 0.159, *n* = 38, *p* = 0.339). In contrast, the difference of the EDC muscle of the left hemisphere was significantly and positively correlated with the illusion of amputation (ρ = 0.409, *n* = 39, *p* = 0.010) and arm ownership (ρ = 0.417, *n* = 39, *p* = 0.008). When removing the 4 outlier points revealed by the ANOVA and repeating the correlation analysis, the results remained the same; only the MEPs’ difference of the EDC muscle of the left hemisphere was significantly and positively correlated with the illusion of amputation (ρ = 0.392, *n* = 38, *p* = 0.015) and arm ownership (ρ = 0.408, *n* = 38, *p* = 0.011). In other words, the stronger the experienced ownership toward the virtual arm and the illusion of amputation, the smaller was the post-VR EDC amplitude compared to the pre-VR EDC amplitude for the left hemisphere (right hand).

## Discussion

The present study investigated whether it is possible for intact individuals to experience the illusion of missing a body-part and whether such perception could yield changes in the excitability of the sensorimotor cortex.

Our results on subjective reports indicate that while participants in both amputation and control conditions experienced the virtual body as their own, similar to previous findings on demographic differences and body ownership (Banakou et al., 2013; Kilteni et al., 2013; Maister et al., 2013; Peck et al., 2013), owning a virtual body of an amputee generated additional sensations of missing the corresponding parts from the real body. Previous studies on body ownership have explored the possibilities of ‘extending’ the participants’ body through the illusion of having a third (Ehrsson, 2009; Guterstam et al., 2011) or a very long limb (Kilteni et al., 2012), or even ‘fading’ it through the illusion of having a phantom body-part (Guterstam et al., 2010; Schmalzl and Ehrsson, 2011; Lewis et al., 2012; Martini et al., 2015). In contrast, the present study asked whether one could experience an illusory ‘reduction’ in the body representation; an amputated virtual body was seen from a 1PP, as if it were spatially coincident with the real body, except for a part of the right arm. Visual and auditory stimuli that emphasized the absence of the body-part were provided: a virtual object was seen to touch the virtual table that is, the former area of the virtual hand, no tactile feedback was provided on the real limb and the collision sound was contextually dissociated from touch on body surface. Our results suggest that through such a disintegration of crossmodal body-related stimuli (i.e., visual, tactile, auditory, and proprioceptive), intact individuals can be made to experience the illusion of missing part of their right arm (Newport and Gilpin, 2011).

Our analysis on the MEP amplitudes and subjective illusion revealed a correlation between the illusion of ownership and amputation with the difference between the pre and post-VR amplitudes; the stronger the illusory experience, the larger the decrease in the EDC corticospinal excitability in the contralateral sensorimotor cortex. How could the amputation illusion be associated with a decrease in corticospinal excitability?

First, previous neurophysiological studies have shown that the corticospinal excitability can significantly change during action observation. For example, when seeing an action, the human motor system is activated in order to ‘resonate’ with the observed action and the excitability of the muscles involved in the action is increased (Fadiga et al., 2005). Therefore, one possible interpretation of our results could be that observing the motor impairments of one’s body (i.e., due to amputation) triggers the motor system to resonate with these limitations and consequently the corticospinal excitability decreases. Nevertheless, this interpretation seems highly unlikely since just seeing an amputated body from a 1PP *per se* was not sufficient to produce excitability changes but it was one’s illusory perception of ownership and amputation that produced the decrease.

Second, an alternative explanation could be based on motor imagery. Imagining an action leads to an enhancement of corticomotor excitability, specifically for the hemisphere that is contralateral to the imagined movement, and for the muscles that are involved in the imagined action (Stinear, 2010). Interestingly, negative motor imagery that is, imagining suppression of hand twitching movements induced by TMS has been shown to reduce the amplitudes of MEPs and suppress the corticospinal excitability (Sohn et al., 2003). This explanation seems unlikely too, since our participants were not instructed to imagine performing any movement but they were explicitly told to remain relaxed and motionless and observe the scene.

In contrast, the pattern of our results tends to support the results from previous neurophysiological studies that investigated the influence of limb deafferentation on the functional reorganization of the motor cortex. Depriving a body area from its sensorimotor information, as for example through immobilization, produces a reduction in the corticospinal excitability of the motor area that is associated with the restricted muscles (Facchini et al., 2002; Huber et al., 2006; Avanzino et al., 2011; Ngomo et al., 2012). Similarly in the present study, we found that experiencing an illusory amputation can yield a decrease in the corticospinal excitability of the EDC muscle and crucially in a correlated fashion; the stronger the illusion of ownership and amputation, the larger the suppression.

Surprisingly, this relationship was not found for the right FDI muscle, a muscle that was clearly included in the amputated area. It is clear that one would expect effects on both right FDI and EDC since both muscles are included in the amputation area. There are possible explanations for the absence of such effect on FDI. First, although participants were explicitly instructed to remain motionless during the experiment, the possibility that they made brief, subtle finger movements that would consequently ‘resurrect’ the missing body-part and eliminate any effect cannot be excluded. An alternative explanation concerns the attention participants paid to specific parts of the virtual body. During most of the VR experimental duration, the virtual ball that was bouncing between the virtual mirror and the former area of the missing body-part was visible, triggering -as a visual moving stimulus- the attention to the forearm rather than the hand (**Figure 1D**). Several studies have found that attention can affect both motor cortex excitability as well as plasticity (Stefan et al., 2004; Conte et al., 2007; Kamke et al., 2012, 2014; Ruge et al., 2014). Attending a specific muscle, influences its corticospinal excitability compared to unattended muscles (Gandevia and Rothwell, 1987). It could be speculated therefore, that the failure to observe changes in the right FDI muscle was due to an increased attention paid to the end of the right forearm rather than the area of the missing virtual hand.

Previous research has provided convergent evidence in favor of a relationship between corticospinal excitability and processing of self-related information. For example, it was observed that during exposure to images containing elements of one’s own face, the excitability of the motor cortex -assessed via TMS- was increased in the right compared to the left hemisphere (Keenan et al., 2001), even when these images were masked (Théoret et al., 2004). In addition, the discrimination between images of self-faces and other-faces was found to be deteriorated when applying repetitive TMS on the right inferior parietal lobule, suggesting again a role of the right hemisphere in processing self-related information (Uddin et al., 2006). Moreover, this modulation in corticospinal excitability of the right hemisphere was found not to be limited to vision of one’s own face, but to extend to vision of one’s own hand and one’s own objects (Salerno et al., 2012). Complementary to these studies, the present research investigated changes in corticospinal excitability as a function of body perception and more particularly of perceived body integrity. Rather than comparing the view of one’s own body (body-part) with viewing somebody else’s body (body-part), we compared two conditions where the viewed body was perceived as one’s own one but was either missing part of its right arm or not. Consistent with this manipulation and in line with the subjective reports of the participants, we found a decrease in the excitability of the left motor cortex (corresponding to the right side of the body) in relation to the illusion of missing part of the right arm and the illusion of owning the viewed right arm.

The present study focused exclusively on MEP amplitudes and it should be noted that analyzing the MEP amplitudes *per se* (i.e., ANOVA) did not reveal any significant differences but just a trend. Future studies should aim at including a larger sample of participants, collecting a larger number of TMS stimuli in order to acquire a stable estimate of corticospinal excitability (Cuypers et al., 2014) and they could fully characterize the nature of these changes, for example by measuring stimulus-response curves or TMS based motor mapping. Moreover, in the present study, only young participants were evaluated. Although, our results cannot be generalized to older population given previous evidence on age effects on MEP amplitudes (Pitcher et al., 2003), we speculate that similar findings would be observed based on the fact that our results are due to the experimental condition and not to the baseline measurement. Finally, although the pattern of our results is similar to that revealed in immobilization studies, the duration of our experimental procedure (13 min) is clearly shorter than that used in those studies (several hours, days, or weeks). Future studies could directly contrast the effects of the body illusion with that of immobilization in order to contrast the size of the effect (magnitude of MEPs’ change), the nature of the effect (including changes in RMTs) as well as its temporal prevalence once the procedure stops.

Finally, previous research on the illusions of body ownership has investigated whether owning a fake hand implies a disownership of the real counterpart. On the basis of a cooling of the real hand as a result of the illusion, some studies have pointed in this direction (Moseley et al., 2008), but others not (Rohde et al., 2013). Assuming that disownership in the case of the RHI were clearly established, it could be argued that in our experiment we have only induced another kind of disownership. However, our experiment concentrated on the absence of the hand, where the real hand was coincident in space first with the intact virtual one, and later when the virtual one was entirely missing. From our results we cannot say whether absence is in any way related to the disownership proposed for the RHI. Second, the results of Llobera et al. (2013) suggest that when the virtual hand is collocated with the real one, and there is full body ownership, there is no disownership of the real hand, but rather a unification of the real and virtual body into one combined entity, which is ‘owned.’ Third, we have shown that when part of the body is missing, there is a decrease in the corticospinal excitability corresponding to that body-part. Whether these physiological changes reflect ‘disownership’ rather than just ‘absence’ is beyond the scope of this paper. However, if they did, then we should have observed similar changes in MEPs for the control group that experienced the same arm and full body ownership but we did not.

To conclude, our study demonstrates the possibility of inhibitory effects on corticospinal pathways, triggered by a short-term illusory perception of missing a body-part and importantly in the absence of any physical intervention (e.g., ischaemic nerve block or movement restriction); just a few minutes of a novel body experience seem to be sufficient to produce direction specific effects on the excitability of sensorimotor system. Although, future studies are needed to investigate whether the origin of the effects is primarily cortical or spinal, as well as their temporal prevalence, our results indicate new possibilities for inducing short-term functional reorganization and plasticity of the sensorimotor system by emphasizing the contribution of body experience. Experience is a known modulator of the brain in both functional and structural terms (May, 2011; Makin et al., 2013) and its role has been widely discussed in relation to increased use, disuse and transient deafferentation of a body-part in healthy participants. A novel perception of the body that suggests that a body-part is missing may also entail psychologically both the body- part’s disuse (inability to move it) and deafferentation (inability to receive afferent input from it), since it is no longer there.

## Author Contributions

KK conceived and designed the experiment with the help of AR-F and MS. KK and JG-S carried out the experiment. The TMS recordings were designed by JG-S, MV, AR-F and carried out by JG-S. The analysis was carried out by KK and MS. The paper was written by KK, MS, AR-F with the help of all the authors, and reviewed by all the authors.

## Conflict of Interest Statement

The authors declare that the research was conducted in the absence of any commercial or financial relationships that could be construed as a potential conflict of interest.

## Supplementary Material

**FIGURE S1 | Residual errors’ plot.** Visual inspection of the plot clearly indicates the existence of four outlier values.
