# Supplementary figures and images for: Decreased Corticospinal Excitability after the Illusion of Missing Part of the Arm
Source: Front Hum Neurosci. 2016 Apr 14;10:145. doi: 10.3389/fnhum.2016.00145 (PMC4830822; doi:10.3389/fnhum.2016.00145)

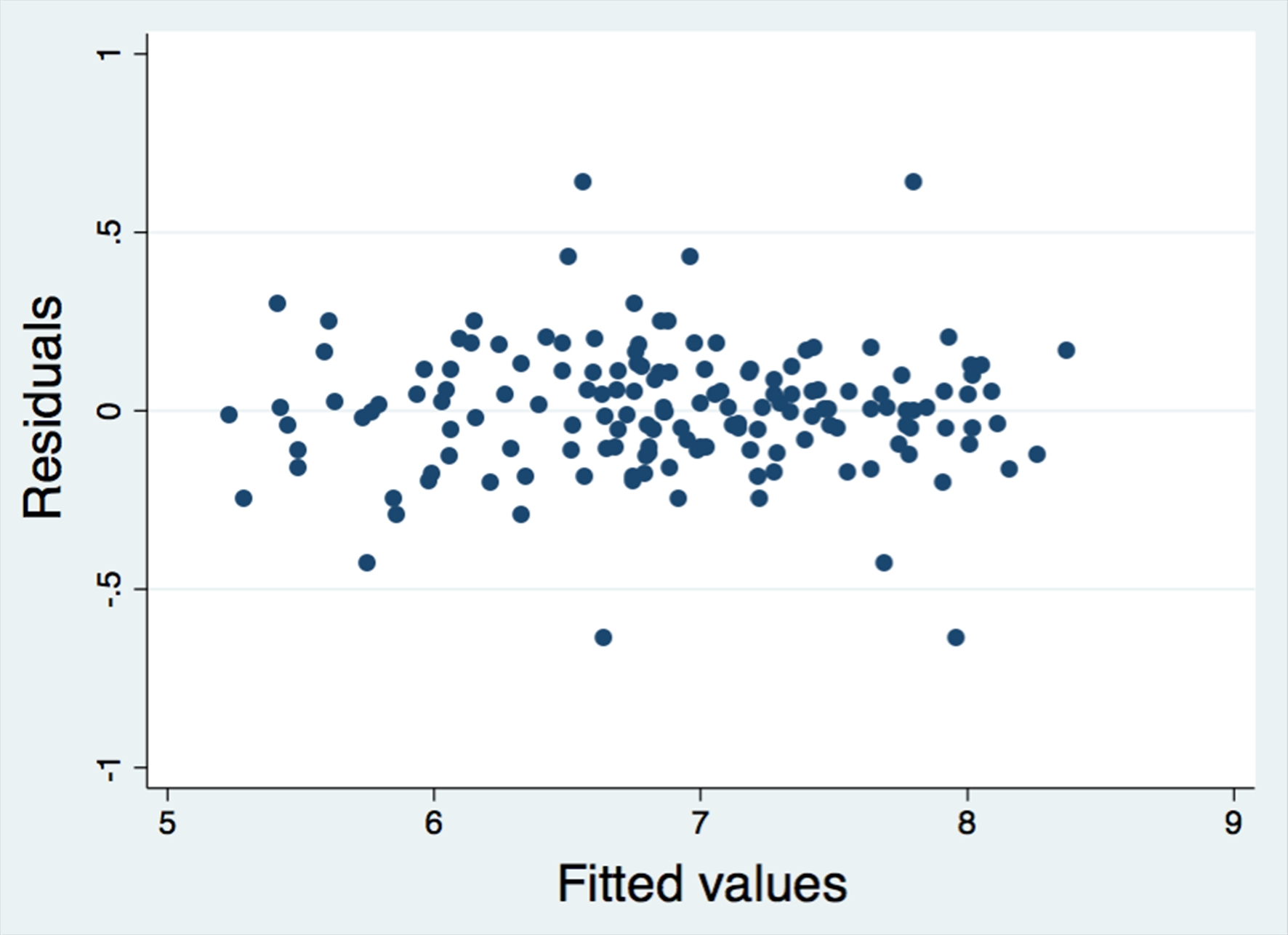

Supplement: Supplementary file 1 [file Image_1.TIF]
